# Disjunction between canola distribution and the genetic structure of its recently described pest, the canola flower midge (*Contarinia brassicola*)

**DOI:** 10.1002/ece3.6927

**Published:** 2020-10-26

**Authors:** Erin O. Campbell, Julian R. Dupuis, Jennifer Holowachuk, Shane Hladun, Meghan A. Vankosky, Boyd A. Mori

**Affiliations:** ^1^ Department of Agriculture, Food, and Nutrition Sciences 4‐10 Agriculture/Forestry Centre University of Alberta Edmonton AB Canada; ^2^ Department of Entomology University of Kentucky Lexington KY USA; ^3^ Agriculture and Agri‐Food Canada Saskatoon Research and Development Centre Saskatoon SK Canada

**Keywords:** agriculture, Cecidomyiidae, Diptera, integrated pest management, population genomics, single nucleotide polymorphisms

## Abstract

Population genomics is a useful tool to support integrated pest management as it can elucidate population dynamics, demography, and histories of invasion. Here, we use a restriction site‐associated DNA sequencing approach combined with whole‐genome amplification (WGA) to assess genomic population structure of a newly described pest of canola, the diminutive canola flower midge, *Contarinia brassicola*. Clustering analyses recovered little geographic structure across the main canola production region but differentiated several geographically disparate populations at edges of the agricultural zone. Given a lack of alternative hypotheses for this pattern, we suggest these data support alternative hosts for this species and thus our canola‐centric view of this midge as a pest has limited our understanding of its biology. These results speak to the need for increased surveying efforts across multiple habitats and other potential hosts within Brassicaceae to improve both our ecological and evolutionary knowledge of this species and contribute to effective management strategies. We additionally found that use of WGA prior to library preparation was an effective method for increasing DNA quantity of these small insects prior to restriction site‐associated DNA sequencing and had no discernible impact on genotyping consistency for population genetic analysis; WGA is therefore likely to be tractable for other similar studies that seek to randomly sample markers across the genome in small organisms.

## Graphical abstract

The canola flower midge is a newly discovered pest of canola from the Canadian Prairies. The population genetic analyses presented in this study recovered little population structure across its known range, consistent with high levels of gene flow in this region. However, a few geographically disparate populations were genetically distinct, and we suggest that these results reflect bleed‐over genetic structure from an unsampled and unknown alternative host range.

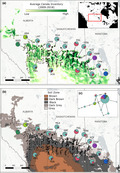

## 1 INTRODUCTION

Population genetics is a powerful tool for integrated pest management and informs effective management strategies by elucidating how genetic diversity, population size, and habitat connectivity influence population dynamics (Combs et al., 2019; Pélissié et al., 2018; Rollins et al., 2006; Tiroesele et al., 2014). Genetic assessments of population dynamics are particularly important when organisms lack comprehensive historical occurrence records (e.g., Mori et al., 2016) or are not easily observed in the field, for example, due to their small size, short life span, or concealed life stages. Population genetics has traditionally utilized gene sequences or microsatellite data for relatively low numbers of markers. However, next‐generation sequencing (NGS) approaches, particularly those that use restriction enzymes to digest DNA and ultimately produce large single nucleotide polymorphism (SNP) datasets, have recently become widespread. These approaches can assess hundreds or thousands of markers across the genome in organisms with no existing genomic resources (Andrews et al., 2016; Davey & Blaxter, 2010) and often provide a more comprehensive representation of population structure compared to one or a few markers (Dussex et al., 2016; Vendrami et al. 2017). Additionally, these SNP‐based datasets can have multifaceted uses in applied pest management settings, such as contextualizing migration routes (Liu, Mori, et al., 2019; Liu, Chen, et al., 2019), providing rapid pathway analysis tools for recurrently invading pests (Picq et al., 2017, Dupuis et al., 2019), and improving existing management tools such as sterile insect technique (Sim et al., 2017).

Despite having many advantages over traditional sequencing approaches for population genetics, one significant technical shortcoming of these restriction enzyme‐based methods is that they require a higher quality and quantity of input DNA than traditional gene or microsatellite sequencing (Andrews et al., 2016; Ballare et al., 2019). Thus, the use of these techniques in exceptionally small‐bodied organisms, such as many insects, has been limited. The development of whole‐genome amplification (WGA) techniques, which amplify genomic DNA prior to NGS library preparation and sequencing, present a possible solution to this problem; however, few studies have assessed whether WGA is likely to introduce amplification biases that may impact genome coverage and genotyping, particularly in small organisms lacking a reference genome (Lovmar and Syvänen, 2006; El Sharawy et al., 2012; Ellegaard et al., 2013; Cruaud et al., 2018).

Flies in the family Cecidomyiidae are typically minute in size, and many species form galls on host plants (Merritt et al. 2009). Several species, notably Hessian fly, *Mayetiola destructor* (Say) and swede midge, *Contarinia nasturtii* (Kieffer), are specialist herbivores and serious agricultural pests (Hallett & Heal, 2001; Lamiri et al., 2001; Liu, Mori, et al., 2019; Liu, Chen, et al., 2019; Schmid et al., 2018), while others, such as the aphid midge, *Aphidoletes aphidimyza* (Rondani), and leafy spurge gall midge, *Spurgia capitigena* (Bremi), have been studied for their potential as biocontrol agents (Boulanger et al., 2018; Lloyd et al., 2005). There have been several population‐level studies of cecidomyiids (for instance, Skuhrava et al., 1984; Black et al., 1990; Lloyd et al., 2005; Redfern & Hunter, 2005; Sato et al. 2020); however, population genetic assessments have typically been limited to only a few molecular markers (e.g., allozymes or gene sequences). While the small size of most cecidomyiids may have initially limited the utility of genome‐wide SNP approaches for population studies, recently developed WGA techniques may make such genomic assessments of these economically important pest and biocontrol species more feasible.

The canola flower midge (CFM), *Contarinia brassicola* Sinclair, is a newly discovered cecidomyiid fly from the Canadian prairies that forms flower galls on canola, *Brassica napus* L. (Mori et al., 2019). Canola was initially developed from rapeseed, *Brassica rapa* L. and *B. napus,* in the Canadian provinces of Manitoba and Saskatchewan in the 1970s and has since increased to become one of the largest oilseed crops in the world due to widespread use as livestock feed, biofuel, and cooking oil (Barthet, 2016; Canola Council of Canada, 2020a). Today, the Canadian Prairies produce and export the largest amount of canola in the world, and the highest levels of Canadian production occur in the province of Saskatchewan (LMC International, 2016; Statistics Canada, 2019).

CFM is hypothesized to be native to Canada due to its documented parasitoid diversity, mitochondrial *COI* diversity, and relatively large range across the Canadian Prairies (Mori et al., 2019), although knowledge of its biology is limited by the short history of its taxonomic recognition. Prior to its description in 2019, the canola midge pests of the Prairie provinces were erroneously thought to be the swede midge, *C. nasturtii*, a morphologically and ecologically similar congener of CFM that was first detected in North America in the eastern Canadian province of Ontario in 2000 (Hallett & Heal, 2001; Canadian Food Inspection Agency 2009). Swede midge causes significant crop damage in parts of Europe, Asia, and more recently, as an invasive pest of canola in North America (Chen et al., 2011; Hallett et al., 2007). In 2007 and 2008, swede midge was first reported from the Canadian Prairies; however, no populations established and all subsequent specimens were later confirmed to be an unknown species, which was later described as CFM (Mori et al., 2019; Soroka et al., 2019). To date, there have been no validated reports of swede midge from the Canadian Prairies and attempts to hybridize the two species in the laboratory have not been successful (BAM, unpublished).

Like swede midge, CFM appears to be multivoltine. Initial adult emergence occurs in June and July, during canola bud formation, with a second generation in August; however, CFM larvae have been observed in the field throughout the summer and into September, suggesting that they may produce more than two generations per year (Andreassen et al., 2018; Chen et al., 2011; Mori et al., 2019; Soroka et al., 2019). Both adults and larvae are small, up to a few millimeters in length, and larvae feed hidden within developing canola flower buds. This causes the buds to transform into galls, which then fail to flower or produce seed (Mori et al., 2019). Due to their feeding behavior and ability to produce multiple generations per year, CFM is potentially capable of causing significant impact on Canadian canola crop yields.

While several aspects of CFM ecology have been described (Mori et al., 2019; Soroka et al., 2019), little is known about CFM population dynamics. Prior genetic investigation of CFM was restricted to specimens sampled primarily from Saskatchewan and use of only a single mitochondrial gene (Mori et al., 2019). There have been no assessments of CFM population structure at larger geographic or genomic scales, thus limiting effective monitoring and risk assessment across the canola‐producing region.

Here, we sampled CFM across its known range in order to assess population genetic structuring using genomic SNPs and a fragment of the mitochondrial *COI* gene. We also investigated whether the use of WGA prior to NGS introduced differences in locus recovery, SNP genotyping, and estimates of polymorphism that may impact downstream population genomic analyses. This is the first population genetic study of CFM, which presents a data‐rich foundation for continued study and highlights several areas for future research to improve risk assessment and monitoring efforts for this species.

## 2 METHODS

### 2.1 CFM surveying, specimen collection, and DNA extraction

A comprehensive survey for CFM was conducted throughout the canola‐producing regions of Alberta, Saskatchewan, and Manitoba in 2017 and 2018 (Vankosky et al., in preparation). Surveyors visited 546 fields from the northern limit of canola production to the southern limit of CFM range in Alberta and Saskatchewan. In Manitoba, the survey was mostly limited to the agricultural extent in the northwest of the province, with the exception of a single, additional site in Portage la Prairie. At each site, 100 canola racemes along the edge of each field were examined. All galled flowers found were collected and returned to the laboratory in a refrigerated container. In the laboratory, buds were dissected and larvae were placed into individual 2 ml tubes and frozen at −80°C. From all survey results, we subsampled sites for genetic analysis by selecting the sites that had the highest CFM densities, defined as any location where more than four larvae were sampled. Our genetic sampling also aimed to maximize the geographic scope across the range of CFM.

Genomic DNA was extracted from whole specimens sampled at 16 localities (Table S1) using a QIAamp DNA Micro Kit (Qiagen). The final DNA concentration of each sample (either with or without WGA, see below) was standardized to 20 ng/µl for library preparation following the two‐enzyme genotyping‐by‐sequencing (GBS) method of Poland et al. (2012).

### 2.2 Whole‐genome amplification, library preparation, and sequencing

Given the small body size of CFM and the relatively high amount of input DNA required for GBS (200 ng per sample), consistently isolating enough DNA from each specimen was challenging. Recently developed WGA methods, such as the REPLI‐g WGA Mini Kit (QIAGEN), hold promise for NGS studies of small organisms. The REPLI‐g Mini Kit uses multiple displacement amplification to amplify genomic DNA (Cheung & Nelson, 1996), and typical usage can produce an average product length of 10 kb. These kits advertise uniform DNA amplification; however, some studies have suggested that they can introduce amplification biases, impacting genome coverage, and they have also been reported to co‐amplify contaminant DNA (Ellegaard et al., 2013; de Medeiros & Farrell, 2018). Although a handful of studies have used such WGA kits for NGS of small organisms (Blair et al., 2015; Cruaud et al., 2018; de Medeiros & Farrell, 2018; Onyango et al., 2015), only two studies have assessed the impact of amplification biases in nonpooled samples of individuals using restriction enzyme‐based SNP genotyping methods, a suite of techniques that includes GBS. Blair et al. (2015) tested the effect of WGA on locus recovery and genotyping using relatively high levels of input DNA (100 ng), per manufacturer's specifications, and reported essentially no difference in locus recovery or genotyping between treatments. A similar study using variable quantities of input DNA (as low as 6 ng) found that genome coverage appeared to be impacted by sample‐specific differences in the amount of DNA used for WGA (de Medeiros & Farrell, 2018).

To test the effect of WGA on GBS sequencing of small insect samples, we created GBS libraries with and without WGA for 24 of the CFM samples collected in 2017 (*n* = 48 libraries). Given preliminary results of these 48 libraries, the remaining 96 CFM samples collected in 2017 and 2018 underwent WGA prior to library preparation. GBS library preparation largely followed Poland et al. (2012) and used *PstI* and *MspI* restriction enzymes to fragment the DNA; these enzymes are commonly used in other insect systems (see, for instance, Erlandson et al., 2019; Lumley et al., 2019; Picq et al., 2017). Any modifications to this protocol are detailed in Erlandson et al. (2019). Paired‐end sequencing was conducted in two runs using an Illumina HiSeq 2500: The 24 individuals used to assess the effect of WGA on GBS library preparation were pooled and sequenced separately from the remaining 96 individuals. A 439 basepair region of the mitochondrial *COI* gene was also amplified for each specimen and sequenced on an ABI 3730xl Sanger sequencer following Mori et al. (2019). All sequencing (GBS and *COI*) was conducted at the National Research Council of Canada Laboratory (Saskatoon, Saskatchewan, Canada).

### 2.3 Data processing and Stacks parameter testing

GBS sequence data were demultiplexed on the Cedar cluster hosted by Compute Canada using the *process_radtags* module in Stacks v. 2.3 (Rochette et al., 2019). Parameter testing following Paris et al. (2017) was conducted on the 24 individuals sequenced with and without the REPLI‐g treatment (herein referred to as the “WGA test dataset”) using the *denovo_map.pl* script to determine the optimal values of the *M* and *n* parameters during subsequent de novo locus construction and SNP calling. The *M* parameter controls the number of mismatches allowed between stacks in the same individual, which represent unique alleles, and the *n* parameter controls the number of mismatches in stacks across individuals as they are merged into loci (Catchen et al., 2011; Rochette et al., 2019). We tested values between 1 and 9 for both parameters. Lower values of *M* and *n* permit fewer mismatches between stacks and, barring exceptionally high levels of natural polymorphism, should be more optimal in regional studies such as this one, where few geographic barriers exist between populations (Paris et al., 2017).

Following the recommendations in Paris et al. (2017), we additionally set the *m* parameter to 3, which controls the minimum allele depth, and used the *r80* principle, a stringent approach to data filtering that retains only loci that are present in 80% of the dataset. When genomic data are assembled de novo, there is risk of constructing loci from contaminant DNA, and some studies have reported that WGA can increase the representation of such contaminants in raw sequence reads (Ellegaard et al., 2013; de Medeiros & Farrell, 2018). However, contaminant DNA, if present, is typically unequally distributed among samples, so using the *r80* parameter should reduce this risk (Paris et al., 2017); de Medeiros and Farrell (2018) found that a similar stringent filtering approach was effective at removing such contaminants from their dataset. We assessed the number of recovered loci, polymorphic loci, and SNPs across each value of *M* and *n* independently for the WGA and non‐WGA sequences in the WGA test dataset to identify any differences in the data that might be attributed to this treatment prior to GBS library preparation.

For CFM population genomic analyses, we processed all the WGA sequences from both sequencing runs together (*n* = 120, herein referred to as the “population genetic dataset”), specified a minimum minor allele frequency of 3%, limited the number of SNPs output per locus to one using the *‐‐write_random_snp* option in the *populations* module of Stacks to reduce genomic linkage, and removed any individuals with more than 50% missing data. *COI* sequences for the same specimens were aligned and quality checked following Mori et al. (2019).

### 2.4 Population genetic analyses

We conducted hierarchical clustering analyses of SNPs for the 16 sampled localities in the population genetic dataset using principal components analysis (PCA) and the program Structure 2.3.4 (Pritchard et al., 2000). Principal component analyses (PCAs) were conducted using *glPca* in adegenet (Jombart, 2008), implemented in R 3.6.1 (R Core Team, 2019), and plotted with ggplot2 (Wickham, 2009). Structure was set to use the admixture model and correlated allele frequencies and was run with and without using sampling locations as a prior (*locprior* vs. *nolocprior*). We tested *K* = 1–20 with 20 independent replicates per value of *K*. Each value of *K* ran for 400,000 MCMC reps with a burn‐in period of 200,000, and we averaged runs using CLUMPAK v1.1 (Kopelman et al., 2015). Following the recommendations of Janes et al. (2017), we considered multiple metrics when determining the optimal value of *K*, including comparison to the PCA, LnPr(*X*|*K*) (Pritchard et al., 2000), Δ*K* (Evanno et al., 2005), and the statistics proposed by Puechmaille (2016). We calculated the latter with StructureSelector (Li & Liu, 2017) using a population map corresponding to collection localities, and a threshold for cluster placement set to 0.5.

SNP pairwise *F*_ST_ was calculated in R using StAMPP (Pembleton et al., 2013) with 1,000 bootstrap permutations and a Benjamini–Hochberg p‐value correction. Expected and observed heterozygosity (*H*_e_ and *H*_o_, respectively) were calculated in dartR (Gruber & Georges, 2019). Isolation‐by‐distance (IBD) analysis using Euclidean distance and a Mantel test with 10,000 permutations was conducted using the R packages sna (Butts, 2019), geosphere (Hijmans, 2019), and adegenet (Jombart, 2008). Due to potentially different biological scenarios impacting the correlation between genetic and geographic distance (e.g., a single genetic cline vs. two or more distinct clines, Maitra et al., 2019; Meirmans, 2012; Teske et al., 2018), the densities between points were visualized with a kernel density estimation function using the package MASS (Venables & Ripley, 2002).

PopART (Leigh & Bryant, 2015) was used to construct a minimum spanning network of *COI* haplotypes.

### 2.5 GIS mapping

To assess whether population genetic structure corresponded to landscape or ecological factors, we used QGIS (QGIS Development Team, 2019) to overlay Canadian canola spatial density and soil zone data (open.canada.ca) on maps depicting the survey locations and average genetic clustering output by Structure for the CFM population dataset. The canola overlay depicts crop inventory values based on satellite imagery (averaged between 2009–2018) as rasters that indicate the level of estimated canola spatial density at each geographic location; regions of green indicate high canola density, and regions of pale yellow represent low density. Yearly canola inventory maps were not available, so we were unable to consider any impact of temporal changes in regional canola inventory on CFM population structure. The soil zone overlay depicts the approximate agricultural extent of the Canadian Prairies and was used to define the northern boundary of the CFM survey (see survey methods above).

## 3 RESULTS

### 3.1 CFM surveys

**FIGURE 1:**
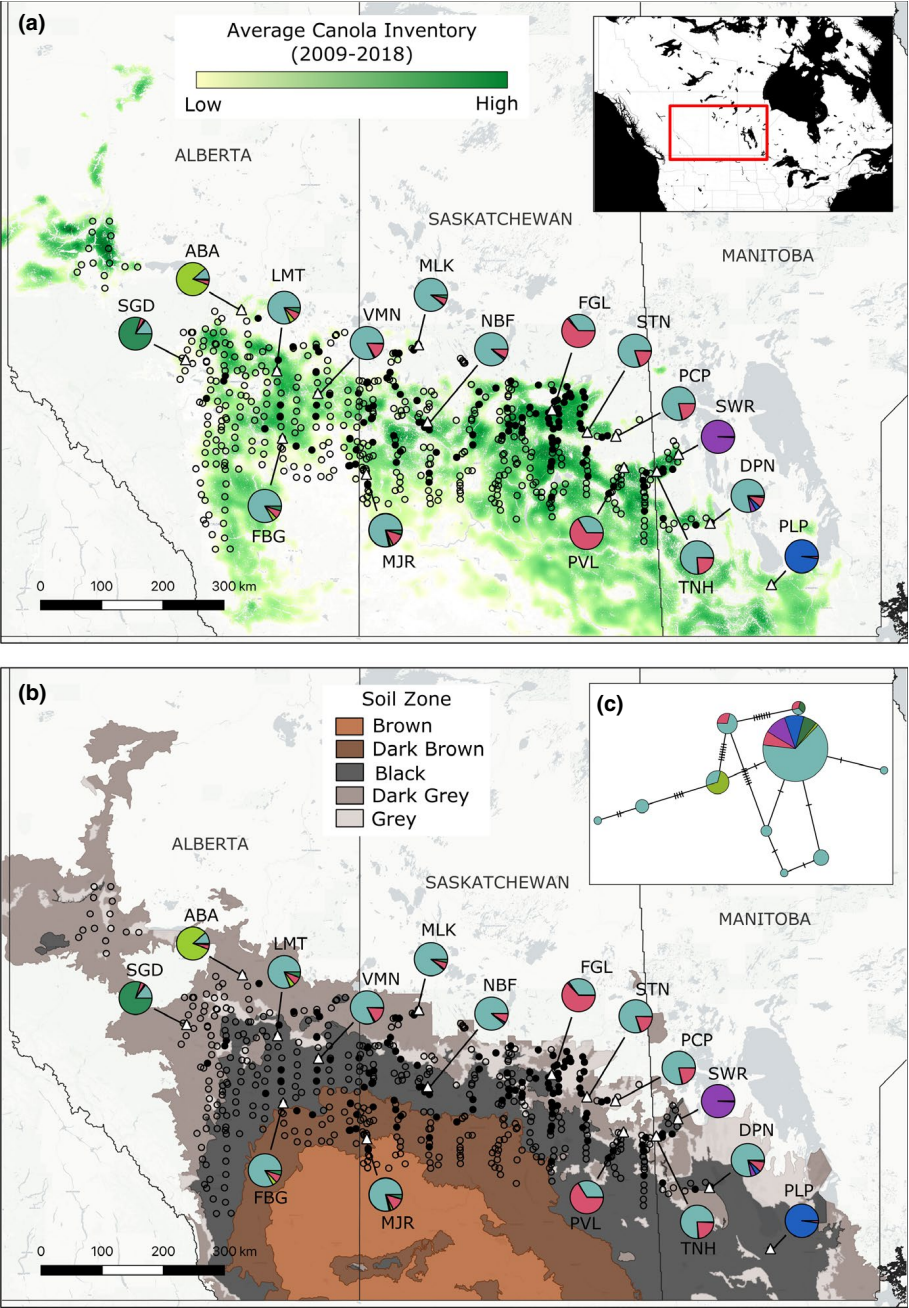
Maps of the Canadian Prairie Region showing canola inventory (a) and soil zones (b). The red box in the inset map (top right) depicts the mapped region in (a) and (b). Pie charts in (a) and (b) correspond to the white triangles plotted on the map (connected by black lines) and depict the average cluster assignments for each population in the CFM population dataset from the *K* = 6 Structure analysis shown in Figure 2d. Panel (c) contains a haplotype map of the COI gene for the same individuals, colored according to their majority cluster assignment from the *K* = 6 Structure analysis. Black, unfilled circles in (a) and (b) represent survey sites where CFM larvae were not found; filled circles indicate sites where larvae were found. Locality names are abbreviated as follows: ABA, Athabasca; DPN, Dauphin; FBG, Forestburg; FGL, Fairy Glen; LMT, Lamont; MJR, Major; MLK, Meadow Lake; NBF, North Battleford; PCP, Porcupine Plain; PLP, Portage la Prairie; PVL, Preeceville; SGD, Sangudo; STN, Steen; SWR, Swan River; TNH, Thunder Hill; VMN, Vermilion

CFM surveys in 2017 and 2018 recovered larvae at 135 of the 547 sites sampled in the northern prairie regions of Manitoba, Saskatchewan, and Alberta (excluding the Peace River Region) (Figure 1), albeit in low numbers (<4) at most sites. Areas with positive larval records broadly corresponded to the black, dark gray, and, to a lesser extent, dark brown soil zones where canola production is the highest (Figure 1a,b, Canola Council of Canada, 2020b). These regions are bordered to the north by parkland or boreal forest, and to the south by drier regions where other Brassicaceae crops, such as mustard, are produced in higher quantities than canola (Diverse Field Crops Cluster, 2020). All of the midges collected were identified as CFM; swede midge was not detected at any of the sites sampled for this study.

### 3.2 Sequence data characteristics and de novo locus construction

#### 3.2.1 WGA test dataset

Samples treated with WGA prior to GBS library preparation had higher numbers of raw sequence reads relative to the non‐WGA samples; however, this read abundance was not evenly distributed across individuals (Table S1). The WGA test dataset (24 individuals sequenced with and without WGA = 48 sublibraries) produced a total of 445.5 million raw sequence reads; 57.2 million reads were attributed to the non‐WGA‐treated sequences and the remaining 388.4 million to the WGA‐treated sequences (Appendix S1: Table A1). After quality filtering, the number of retained reads dropped to 8.7 million and 80.4 million, respectively. Approximately 68% of the total sequencing reads were discarded during quality filtering due to adapter contamination, while only 2.2% of the total reads were discarded due to low quality. Across samples, 8 of the 24 samples represented approximately 80% of the WGA raw sequence reads (min: 21.6 million, max: 70.3 million, mean: 38.9 million, Appendix S1: Table A2). The remaining 16 samples contained markedly fewer raw sequencing reads (min: 2.7 million, max: 8.7 million, mean: 4.8 million). While the non‐WGA samples had a more even distribution of raw reads across samples, the same proportion of samples (8 of 24) still contained the majority (55%) of the non‐WGA raw reads (min: 2.9 million, max: 6 million, mean: 3.9 million, Appendix S1: Table A2), and 5 of these highly sequenced individuals were the same between treatments.

Next, we assessed the number of invariant loci, polymorphic loci, and SNPs for each tested value of *M* and *n* using the 48 libraries in the WGA test dataset (24 with WGA and 24 without). Following Paris et al. (2017), we chose parameter values for *M* and *n* that optimized both the number of polymorphic loci and SNPs, and for both the WGA and non‐WGA treatments these values were maximized at *M*2*n*2. In the resulting dataset, we observed large differences in the number of polymorphic loci, SNPs, and overall read depth between the two treatments. The non‐WGA samples had more than twice the number of loci and SNPs than the samples treated with WGA, and the mean depth of coverage in these sequences was approximately 30% that of the WGA samples (Appendix S1: Table A3). However, the mean number of SNPs per locus between treatments (non‐WGA = 2.4, WGA = 2.1, Table A3) and values of observed heterozygosity (non‐WGA = 0.15, WGA = 0.13, Table A3) were similar. Additionally, pairwise *F*_ST_ calculations between the WGA and non‐WGA sequences for each population were zero (Appendix S1: Table A4), and a PCA of this dataset clustered libraries by sample, not WGA treatment (Appendix S1: Figure A1).

#### 3.2.2 Second sequencing run and population genetic dataset

The second sequencing run (96 individuals treated with WGA prior to sequencing) produced a total of 354.9 million sequence reads, which was reduced to 69.3 million after quality filtering; here, 70.7% of sequence reads were removed during quality filtering due to adapter contamination, and 1.1% were discarded due to low quality (Appendix S1: Table A1). Both the 24 WGA libraries from the WGA test dataset and these 96 libraries were used to create the population genetic dataset; however, 14 individuals containing more than 50% missing data were additionally removed; after filtering, this dataset contained 106 individuals and 1,702 SNPs (Appendix S1: Table A3) and was used for all subsequent SNP analyses.

### 3.3 SNP population genomic analyses

**FIGURE 2:**
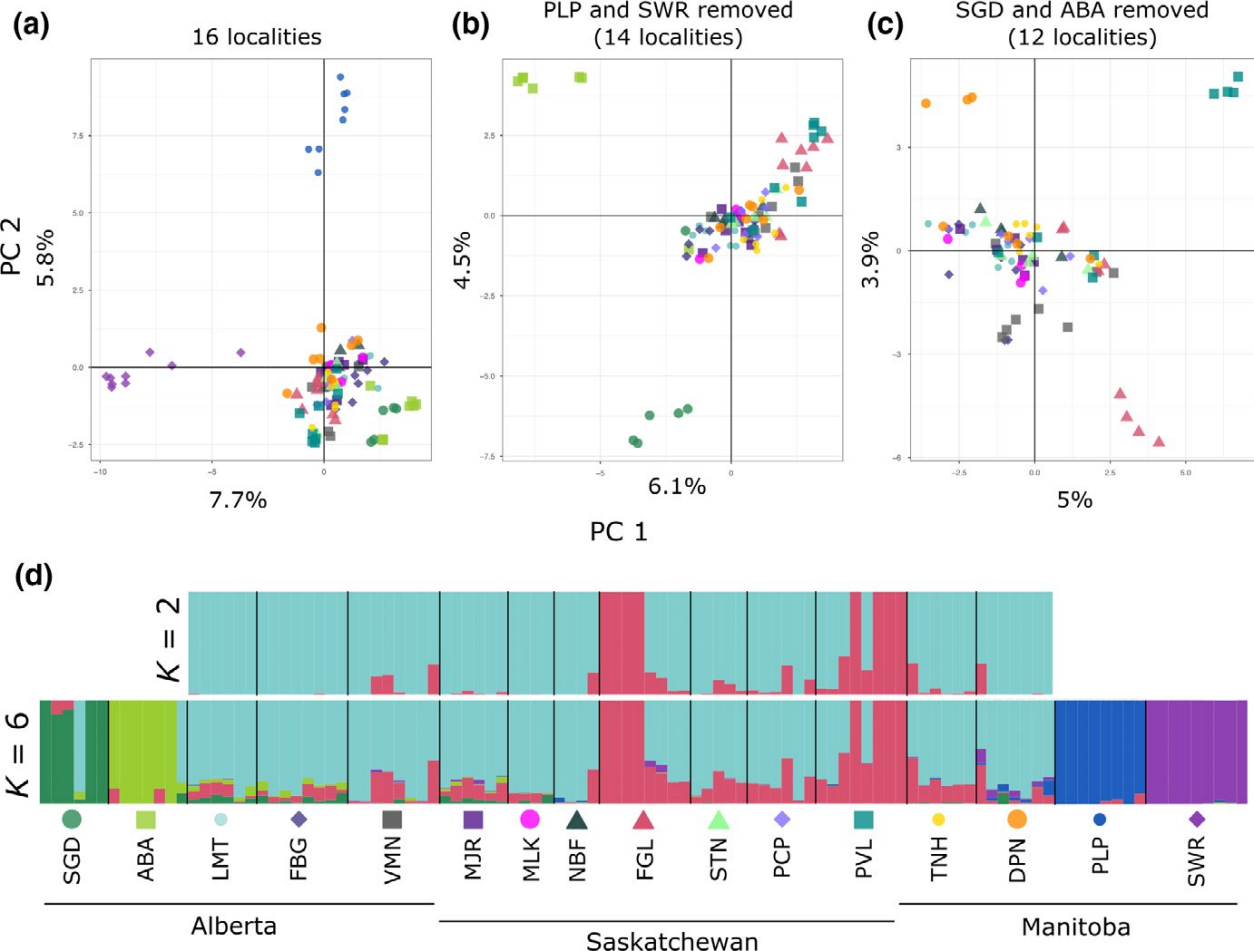
Hierarchical principle component (a‐c) and structure (d) analysis results for the CFM population dataset using genomic SNPs. Colored symbols above the locality abbreviations in (d) correspond to the symbols used for each sampling locality in the PCAs (a‐c). Locality abbreviations follow Figure 1

Results of PCA and Structure were concordant and supported hierarchical population structure within this dataset. In the PCA, the first and second principal components (PCs) of the 16 localities recovered two highly divergent populations from the eastern edge of the sampled region in Manitoba: Swan River and Portage la Prairie (Figure 2a). Two Albertan localities on the western edge of our sampling region, Athabasca and Sangudo, were less distinct but the combined effect of PC 1 and PC 2 clustered them apart from the remaining 12 central localities. These western and eastern sampling edges broadly coincide with the boundaries of canola production in the Canadian Prairies, excluding the Peace River Region of Alberta, a geographically disparate region in the Boreal Plains northwest of the rest of the prairies (westernmost cluster of survey points in Figure 1); we did not recover any CFM larvae from this region in our 2017 or 2018 surveys. Hierarchical PCA omitting the divergent Manitoba localities (i.e., “14 localities”) separated the two aforementioned western Alberta localities along PC 1 and PC 2 (Figure 2b). Further hierarchical PCA omitting the divergent Manitoba and Alberta localities (i.e., “12 localities”) recovered little additional substructure, although three localities, Fairy Glen, Preeceville, and Dauphin, had some individuals that appeared to be genetically distinct along PC 1 and PC 2 and others that clustered with the remaining central localities (Figure 2c).

In Structure analyses, the use of sampling location as a prior (*locprior*) did not produce substantial differences in cluster assignments when compared to the analyses that did not incorporate this information (*nolocprior*); thus, we focus only on the latter here. We found variable support for an optimal value of *K*: LnPr(*X*|*K*) displayed only a gradual plateau starting at *K* = 5 to 7, Δ*K* values were generally low (maximum Δ*K* = 21.8) but supported *K* = 2, 5, 7, and 9, and the Puechmaille statistics supported *K* = 5, 6, and 7 (Figure S1). Visualization of bar charts for all values of *K* indicated hierarchical structure that matched the results of the PCA: *K* = 2 and 3 separated the two easternmost Manitoba localities, and *K* = 4 separated the two westernmost Alberta localities. At *K* = 5 and 6, some individuals from two Saskatchewan localities (Fairy Glen and Preeceville) formed a distinct cluster, as was observed in the PCA (Figure 2). Beyond *K* = 6, there was little meaningful structure and additional clusters were generally represented by low *Q*‐ratios (all bar charts presented in Figure S1). Additionally, independent hierarchical Structure analyses of the large central cluster (12 localities) supported the same divisions as the *K* = 6 results (Figure 2d, Figure S1), further supporting *K* = 6 as the optimal value of *K*. Finally, two specimens sampled in Sangudo and Athabasca clustered with the central population rather than with their collection locality and likely represent migrants (Figure 2d).

**FIGURE 3:**
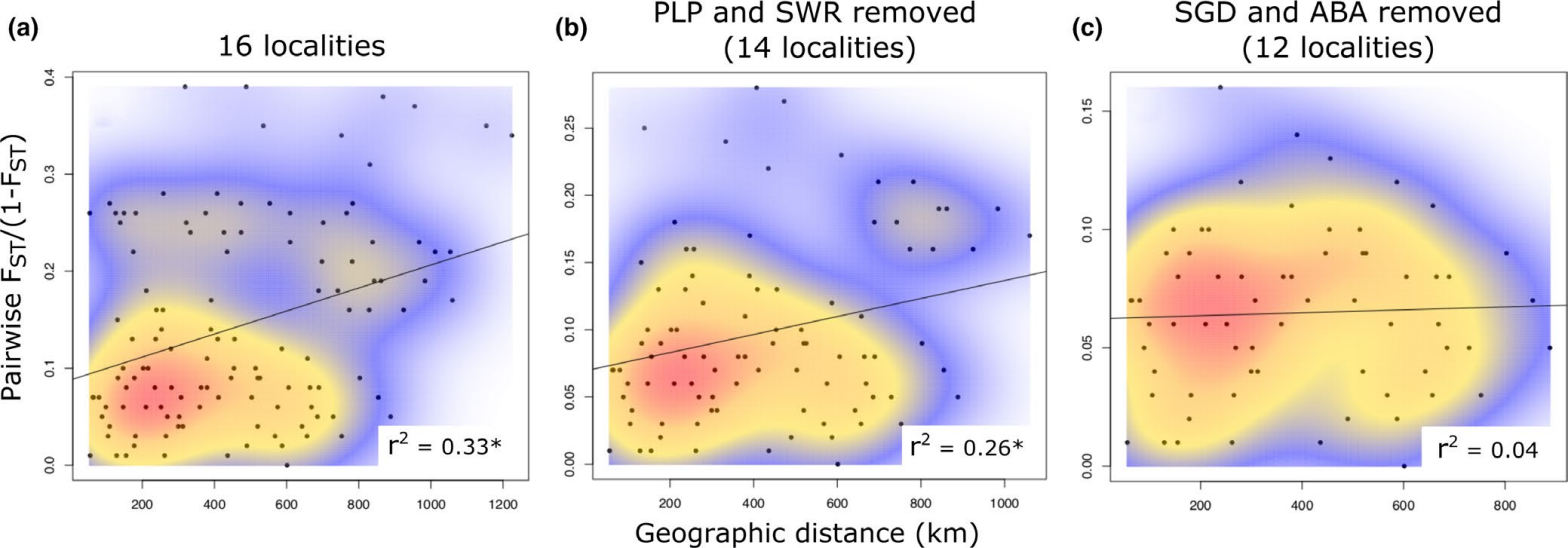
Isolation‐by‐distance (IBD) analyses of SNPs for the 16 sampled populations in the CFM population dataset. Asterisks beside the r^2^ values indicate *p*‐values < .05, and kernel density heatmaps behind the points visualize the “islands” of data points driving the significant results in (a) and (b); after removing these divergent populations, IBD analysis was no longer significant (c). Locality abbreviations follow Figure 1

IBD analysis using Euclidean distance and pairwise *F*_ST_/(1–*F*_ST_) values for all 16 localities was highly significant (*r* ^2^ = .33, *p*‐value = .004, Figure 3a) and remained significant after removing the eastern Portage la Prairie and Swan River localities (*r* ^2^ = .26, *p*‐value = .03, Figure 3b). However, pairwise point densities indicated “islands” of data points rather than a single cline tracking the regression line as would be expected if genetic divergence increased linearly with geographic distance. After additionally removing the Sangudo and Athabasca localities, IBD analysis of the remaining 12 central localities was not significant (*r* ^2^ = .04, *p*‐value = .38, Figure 3c), suggesting that the four divergent localities were the primary drivers of the aforementioned relationships.

Values of expected and observed heterozygosity were moderate and generally similar within each population, except for Swan River and Portage la Prairie, which both had heterozygote excess (*H*_o_ = 0.24, *H*_e_ =0.16 in both populations, Table 1), and North Battleford, which had lower observed values of heterozygosity (*H*_o_ = 0.14, *H*_e_ = 0.21). We note however that the North Battleford population had far higher levels of missing data than the other populations (average missing data of North Battleford population = 45%; average missing data across remaining populations = 9%). Pairwise *F*_ST_ values ranged from 0 to 0.39 (Table 1), and were lower between the 12 central populations (0–0.17) and higher in comparisons including at least one of the four divergent populations (Swan River, Portage la Prairie, Sangudo, and Athabasca) recovered in the PCA and Structure analyses (0.13–0.39).

**TABLE 1:** Population‐level summary statistics

Population	Pairwise *F*_ST_															*N*	Heterozygosity	
	SGD	ABA	LMT	FBG	VMN	MJR	MLK	NBF	FGL	STN	PCP	PVL	TNH	SWR	DPN		*Ho*	*He*
SGD	–															6	0.24	0.17
ABA	**0.25**	–														7	0.22	0.17
LMT	**0.13**	**0.15**	–													6	0.21	0.21
FBG	**0.13**	**0.14**	**0.01**	–												8	0.22	0.23
VMN	**0.16**	**0.18**	**0.05**	**0.04**	–											8	0.23	0.22
MJR	**0.13**	**0.17**	0.01	**0.02**	**0.03**	–										6	0.23	0.20
MLK	**0.22**	**0.24**	**0.05**	**0.04**	**0.06**	**0.03**	–									4	0.16	0.15
NBF	**0.27**	**0.28**	**0.05**	**0.08**	**0.10**	**0.08**	**0.10**	–								4	0.14	0.21
FGL	**0.21**	**0.23**	**0.09**	**0.09**	**0.09**	**0.08**	**0.12**	**0.16**	–							8	0.20	0.20
STN	**0.16**	**0.18**	0.00	**0.02**	**0.04**	0.01	**0.06**	**0.07**	**0.07**	–						5	0.22	0.21
PCP	**0.16**	**0.18**	**0.03**	**0.04**	**0.06**	**0.02**	**0.07**	**0.08**	**0.09**	**0.01**	–					6	0.21	0.20
PVL	**0.19**	**0.21**	**0.08**	**0.08**	**0.08**	**0.07**	**0.13**	**0.14**	**0.09**	**0.06**	**0.07**	–				8	0.21	0.20
TNH	**0.16**	**0.19**	**0.03**	**0.05**	**0.06**	**0.03**	**0.10**	**0.10**	**0.08**	0.01	**0.03**	**0.07**	–			6	0.21	0.21
SWR	**0.37**	**0.38**	**0.27**	**0.26**	**0.25**	**0.26**	**0.35**	**0.39**	**0.28**	**0.26**	**0.26**	**0.27**	**0.26**	–		9	0.24	0.16
DPN	**0.17**	**0.19**	**0.05**	**0.07**	**0.09**	**0.05**	**0.11**	**0.12**	**0.11**	**0.04**	**0.06**	**0.10**	**0.06**	**0.26**	–	7	0.21	0.21
PLP	**0.34**	**0.35**	**0.22**	**0.22**	**0.23**	**0.23**	**0.31**	**0.34**	**0.27**	**0.24**	**0.24**	**0.26**	**0.25**	**0.39**	**0.22**	8	0.24	0.16

Note

Lower diagonal indicates pairwise *F*_ST_ values. *F*_ST_ calculations with *p*‐values < .05 after Benjamini–Hochberg false discovery rate correction are bolded. *N* = sample size; *H*_o_ = observed heterozygosity; *H*_e_ = expected heterozygosity. Population abbreviations are based on the first 3 letters of each population in Figure 1.

### 3.4 COI haplotype mapping and summary statistics

Due to missing nucleotide (nt) sequence at the 5ʹ and/or 3ʹ ends in 20 specimens (min. missing = 7 nt, max. missing = 80 nt, Table S1), we created a masked dataset using the modal sequence of those missing regions for each collection locality to ensure haplotype mapping was not biased by missing data. Two specimens additionally failed to sequence and were omitted from the *COI* dataset (final *n* = 104). The minimum spanning haplotype network depicted a single large haplogroup and nine additional smaller haplogroups (Figure 1c). Central populations (indicated by light blue and pink colors) had the greatest amount of haplotype diversity; however, overall haplotype variation was low (number of segregating sites = 16, number of parsimony‐informative sites = 13), and there was no clear spatial relationship to haplotype variation; except for the Swan River and Portage la Prairie populations, each population had sequences in more than one haplogroup. The Swan River and Portage la Prairie haplotypes were identical and clustered in the large haplogroup with several specimens collected from central populations and the western Sangudo population. The Athabasca population was moderately distinct and clustered mostly in a smaller haplogroup along with a few other specimens from central populations.

## 4 DISCUSSION

### 4.1 Population structure of CFM in the Canadian Prairies

We found little overall geographic structuring related to either canola density or soil zone in the 16 populations included in this study, although nuclear SNP analyses recovered substantially more population structure than *COI* haplotype analysis (Figure 1). Both PCA and Structure analyses using SNPs recovered only four markedly divergent populations (Swan River and Portage la Prairie in Manitoba, and Athabasca and Sangudo in Alberta), located near the edges of canola production in those regions (Figures 1 and 2). While this may be indicative of an edge effect (sensu Cook, 1961), other populations were also sampled near the edges of canola production but were not genetically distinct. The Portage la Prairie population is a possible exception to this, as these individuals were sampled from a research farm (Canada‐Manitoba Crop Diversification Centre) located in the city of Portage la Prairie, and as a result may have reduced opportunities for gene flow with other CFM populations located on more rural farmland.

The remaining 12 central populations formed a large genetic cluster spanning eastern Alberta, Saskatchewan, and western Manitoba. Within this central cluster, Structure analysis indicated two distinct sources of genetic ancestry that were not clearly related to sampling geography (Figures 1 and 2d), and which was particularly pronounced in the Fairy Glen and Preeceville populations. Pairwise *F*_ST_ was also low between these central populations (Table 1), and IBD analysis was nonsignificant (Figure 3c) suggesting few geographic barriers to gene flow. This is consistent with the homogenous landscape throughout much of the Canadian Prairies and the high level of canola inventory in the sampling region of this study (Figure 1a).

*COI* haplotype diversity was relatively low overall, and the four divergent populations in the SNP‐based analyses were not distinct for *COI*. These results are consistent with contemporary, widespread gene flow facilitated by large‐scale canola production in the Canadian Prairies. It is possible that differences in recovered population structure between SNPs and *COI* are due to temporal differences in habitat connectivity resulting from year‐over‐year changes in canola inventory, and/or changes in effective population sizes of CFM due to regional and temporal differences in insecticide use. The *COI* gene represents only a single haploid marker, and if our sampling coincided with a period of greater effective population size and connectivity, we may expect to have less population structure in one or a few markers compared to thousands of diploid nuclear SNPs (Dussex et al., 2016; Liu, Mori, et al., 2019; Liu, Chen, et al., 2019). Data for historical year‐over‐year canola inventory production numbers or insecticide spray records are unavailable over this broad geographic range, so we cannot test this hypothesis at this point in time.

### 4.2 Canola myopia

This study provides a much‐needed foundation for understanding the population genetics and demography of CFM. However, we still know little about the historical ecology and evolution of this species, or whether CFM is likely to be a significant risk to canola production in North America. Notably, the hypothesis that CFM is native, based on its widespread distribution as well as its mitochondrial DNA and parasitoid diversity (Mori et al., 2019), remains speculative. The disjunct distributions of highly differentiated population genetic units in canola‐producing regions may provide additional evidence for this speculation and lines of reasoning for future research.

Our surveying and sampling were limited to canola production regions across the Prairie provinces. Given the short history of widespread canola production in Canada (ca. 40 years), if CFM is native then it must have some native (and/or naturalized) hosts within or outside of this geographic region. Alternative host associations have yet to be thoroughly evaluated for this species, although CFM larvae and galls were found on mustard (*Brassica juncea* va. Centennial Brown) grown in a small plot on an AAFC research farm in Melfort, Saskatchewan (BAM, unpublished). This locality is outside of the typical mustard growing region of southwestern Saskatchewan and inside the primary distribution of CFM. If alternative hosts do exist for this species, our canola‐centric sampling may have anthropogenically biased our assessments of population structure in two ways: (1) these geographically disparate, differentiated populations at the edge of the canola production region may represent bleed‐over genetic structure from an alternative and more geographically widespread host range, and (2) the lack of strong differentiation in the majority of our central localities may reflect a relatively recent bottleneck onto the anthropogenic host.

Saskatchewan and parts of southwestern Manitoba were the first regions to cultivate canola in Canada and account for the majority of canola yield worldwide (Barthet, 2016; Statistics Canada, 2019). The first confirmed observations of CFM were also from this region (Soroka et al., 2019). If CFM is native, as hypothesized, it is possible that the lack of population structure recovered in the central localities is reflective of a recent population expansion in this region after a host switch event that likely occurred shortly after canola was established in the Canadian Prairies. Furthermore, due to our canola‐centric sampling, our current assessment of population structure may suffer from the presence of unsampled “ghost populations” (sensu Beerli, 2004) present on alternative hosts both within and outside of the canola production region. This may at least partially explain the genetic distinctiveness of the Sangudo, Athabasca, Portage la Prairie, and Swan River populations relative to each other and to other, nearby populations, as well as the substructure recovered in our clustering analyses (Figures 1 and 2) and the ambiguous support for an optimal value of *K* in Structure analyses (Figure S1); failing to sample ghost populations can decrease confidence in population assignments of sampled individuals (Beerli, 2004; Slatkin, 2004). This is largely supposition at this point; however, given the lack of alternative hypotheses to explain the disjunct pattern of highly differentiated populations at the edges of the canola production region, we believe it deserves additional scrutiny and research effort.

### 4.3 Whole‐genome amplification and GBS sequencing performance

We observed differences in sequencing coverage between treatments in the WGA test dataset that may be attributed to multiple factors. Five of the eight most highly sequenced samples were the same between the WGA and non‐WGA treatments, so those specimens may have had higher initial molecular weight DNA compared to the other 16 individuals, which could result in more sequence tags being cut and amplified (Andrews et al., 2016). However, this does not sufficiently explain the overall greater number of sequence reads attributed to the WGA samples. Perhaps most significantly, we observed a high level of adapter contamination in both sequencing runs, regardless of WGA treatment. This is generally the result of input DNA fragments being shorter than the 150 bp sequencing length, thus leading to adapter sequence integration into the 3ʹ ends of the sequencing reads and subsequent sequencing of these regions (Illumina, 2020). Bioanalyzer results for the WGA test and population genetic datasets confirmed that a high proportion of short insert fragment lengths were present in our final libraries (shorter than 150 bp excluding sequencing adapters, results not shown). Despite this, after processing the retained sequence reads using Stacks, we were successful in assembling a moderate number of loci with sufficient read depth for population genomic analyses (Appendix S1: Table A3). Thus, while a greater number of useable sequencing reads would have likely increased the overall number and depth of retained loci, this contamination does not appear to have compromised the study, analytically.

Our results also indicate a trade‐off between sequencing coverage and read depth when using WGA prior to GBS (Appendix S1: Table A3). This is concordant with the findings of de Medeiros and Farrell (2018), who found that samples with less input DNA were more prone to reduced genome coverage after sequencing. Our results differ from those of Blair et al. (2015) and Cruaud et al. (2018), who both found negligible differences in genome coverage and sequencing depth when comparing WGA and non‐WGA samples. However, we note that Blair et al. (2015) used much higher quantities of input DNA for WGA than our study system permitted, and Cruaud et al. (2018) pooled individuals so they were unable to make the same individual comparisons presented here and in de Medeiros and Farrell (2018).

Reported differences in sequencing depth between treatments did not appear to impact de novo locus construction and SNP calling in the WGA test dataset, which was consistent with other studies (Blair et al., 2015; Cruaud et al., 2018; de Medeiros & Farrell, 2018). Pairwise *F*_ST_ comparisons, observed heterozygosity, and PCA indicated little difference in genotyping between treatments when they were filtered together (Appendix S1: Table A3, A4; Figure A1). Our results suggest that, despite the potential for unequal amplification of genomic DNA by WGA, this approach is not likely to produce significant biases that impact downstream de novo SNP calling, provided that read depth is sufficient. Therefore, we suggest that the benefits of WGA (namely, facilitating the use of single specimens of small species for NGS) in studies that seek to randomly sample markers across the genome outweigh the potential shortcoming of reduced genome coverage.

## 5 CONCLUSIONS

Here, we present the first genetic assessment of population structure for CFM and additionally used WGA to generate GBS libraries from these small insects. Although we found some impact of WGA on the resulting raw sequence data, there was no appreciable impact on filtered datasets and subsequent population genomic analyses.

Overall, the GBS dataset recovered little population structure across the majority of the sampled CFM populations, although much more so than the comparable mitochondrial dataset. The only strongly differentiated populations were geographically disparate and located at the edges of the canola production region. Given a lack of alternative explanations for this pattern, we expounded on the hypothesis that CFM is a native species that has unrecognized hosts both within and outside of the main agricultural zone, which is where research on this newly described species has focused thus far. Therefore, it will be vital to increase survey efforts to other Brassicaceae both within and outside canola production regions in future studies.

Expanded surveying to include more diverse potential habitats will provide important information about the range and host preferences of this species and facilitate comparisons of regional or host‐associated population densities that may inform CFM risk assessments and monitoring. Temporal sampling throughout the growing season will also help to clarify the number of generations that CFM can produce each year and elucidate the ecological and population dynamics of early versus late generations. Finally, if our hypothesis of alternative hosts is substantiated, CFM may provide a unique model system for studying the consequences of a contemporary host shift onto a major commercial crop species, thus informing both the evolution of insect–plant relationships and impacts on pest management (Bernal et al., 2019; Bernal & Medina, 2018; Chen, 2016).

## CONFLICT OF INTERESTS

None declared.

## AUTHOR CONTRIBUTION

**Erin O. Campbell:** Data curation (lead); Formal analysis (equal); Investigation (equal); Writing‐original draft (lead); Writing‐review & editing (equal). **Julian R. Dupuis:** Formal analysis (equal); Investigation (equal); Writing‐original draft (supporting); Writing‐review & editing (equal). **Jennifer Holowachuk:** Investigation (equal); Methodology (equal); Writing‐review & editing (supporting). **Shane Hladun:** Investigation (equal); Methodology (equal); Writing‐review & editing (supporting). **Meghan A. Vankosky:** Conceptualization (equal); Funding acquisition (equal); Investigation (equal); Methodology (equal); Writing‐review & editing (supporting). **Boyd A. Mori:** Conceptualization (equal); Formal analysis (equal); Funding acquisition (equal); Investigation (equal); Methodology (equal); Writing‐original draft (supporting); Writing‐review & editing (equal).

## Supporting information

Figure S1

Table S1

Appendix S1

## Data Availability

Raw, demultiplexed GBS sequencing files are available on NCBI SRA under BioProject PRJNA656320, accession IDs SRX8961930–SRX8692062, and mitochondrial DNA sequences are available on GenBank, accession IDs MT554712–MT554815.
