# Supplementary material for: Disjunction between canola distribution and the genetic structure of its recently described pest, the canola flower midge (Contarinia brassicola)
Source: Ecol Evol. 2020 Oct 26;10(23):13284–96. doi: 10.1002/ece3.6927 (PMC7713945; doi:10.1002/ece3.6927)
Supplement: Supplementary file 1 — Figure S1 [file ECE3-10-13284-s001.pdf]

Fig. S1: Full Structure results for all individuals (left) and 12 localities (right)

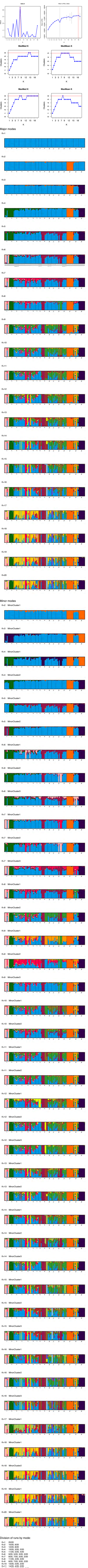

Division of runs by mode:

K=1 20/20
K=2 10/20, 9/20
K=3 12/20, 8/20
K=4 13/20, 3/20, 2/20
K=5 11/20, 5/20, 4/20
K=6 9/20, 5/20, 3/20, 3/20
K=7 8/20, 7/20, 3/20, 2/20
K=8 11/20, 4/20, 3/20
K=9 8/20, 7/20, 3/20, 2/20
K=10 13/20, 5/20, 2/20
K=11 14/20, 4/20, 2/20
K=12 11/20, 3/20, 3/20, 2/20
K=13 8/20, 8/20, 4/20
K=14 8/20, 6/20, 3/20, 2/20
K=15 12/20, 4/20, 2/20, 2/20
K=16 8/20, 6/20, 3/20, 3/20
K=17 16/20, 2/20
K=18 8/20, 6/20, 6/20
K=19 18/20, 2/20
K=20 12/20, 7/20

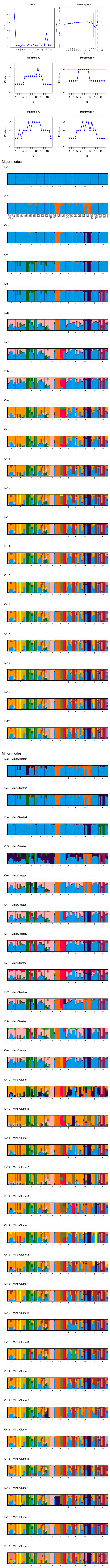

Division of runs by mode:

K=1 20/20
K=2 20/20
K=3 11/20, 9/20
K=4 10/20, 6/20, 4/20
K=5 10/20, 10/20
K=6 17/20, 2/20
K=7 5/20, 5/20, 4/20, 4/20, 2/20
K=8 15/20, 3/20
K=9 15/20, 3/20
K=10 13/20, 4/20, 2/20
K=11 8/20, 6/20, 3/20, 3/20
K=12 13/20, 4/20, 2/20
K=13 13/20, 2/20, 2/20, 2/20
K=14 14/20, 4/20, 2/20
K=15 9/20, 7/20, 2/20
K=16 16/20, 3/20
K=17 14/20, 4/20
K=18 18/20
K=19 17/20, 2/20
K=20 10/20, 8/20
