# Supplementary material for: Disjunction between canola distribution and the genetic structure of its recently described pest, the canola flower midge (Contarinia brassicola)
Source: Ecol Evol. 2020 Oct 26;10(23):13284–96. doi: 10.1002/ece3.6927 (PMC7713945; doi:10.1002/ece3.6927)
Supplement: Supplementary file 3 — Appendix S1 [file ECE3-10-13284-s003.docx]

**Appendix 1**

Table A1: *process_radtags* output summary for all specimens, and a comparison of the number of loci, SNPs, and read depth between specimens sequenced with and without WGA (the same 24 individuals were used for both treatments in the first sequencing run).

|  | Raw sequence reads | | | | | | | | | Retained reads after trimming and quality filtering | | | | | | |  |
| --- | --- | --- | --- | --- | --- | --- | --- | --- | --- | --- | --- | --- | --- | --- | --- | --- | --- |
| Sequencing Run | *N* | Sample prep | total | min | max | mean | reads of low quality (%) | reads with adapter contamination (%) | | total | | min | | max | | mean | |
| 1 (48-plex) | 24 | no WGA | 57,165,942 | 626,106 | 6,043,348 | 2,381,914 | 1.3 | 68* | | 8,713,812 | | 102,880 | | 657,396 | | 363,076 | |
|  | 24 | with WGA | 388,383,448 | 2,703,818 | 70,302,382 | 16,182,644 | 3.1 |  |  | 80,395,074 | | 74,248 | | 1,941,327 | | 3,349,795 | |
| 2 (96-plex) | 96 | with WGA | 354,965,296 | 584,932 | 29,183,536 | 3,613,080 | 1.1 | 70.7 | | 69,263,407 | | 153,642 | | 4,806,709 | | 721,494 | |
|  |  |  |  |  |  |  |  | |  |  |  | |  | |  | |  |
|  | * numbers by treatment were not available, *process_radtags* only outputs the total per run for this metric | | | | | | | | |  |  | |  | |  | |  |

Table A2: Comparison of sequencing output for WGA and non-WGA samples in the “WGA test” dataset prior to locus construction and filtering in Stacks 2.

| Treatment | Group | Min. raw reads | Max. raw reads | Mean raw reads | Min. retained reads | Max. retained reads | Mean retained reads | Mean proportion retained reads |
| --- | --- | --- | --- | --- | --- | --- | --- | --- |
| WGA | 8 most highly sequenced | 21,577,086 | 70,302,382 | 38,953,950 | 4,454,158 | 10,418,879 | 7,064,783 | 20% |
|  | remaining 16 | 2,703,818 | 8,726,628 | 4,796,990 | 889,494 | 2,903,726 | 1,492,301 | 31.20% |
|  |  |  |  |  |  |  |  |  |
| Non-WGA | 8 most highly sequenced | 2,887,374 | 6,043,348 | 3,922,795 | 343,877 | 657,396 | 504,543 | 13.70% |
|  | remaining 16 | 626,106 | 2,693,250 | 1,611,474 | 102,880 | 425,647 | 292,342 | 19.10% |

Table A3: Summary information for filtered datasets (*M2n2*, *r80*). Due to poor sequencing, three individuals in the “WGA test” dataset did not pass filtering parameters and were removed from the dataset (n=21, see also Fig. A1). The population genetic dataset (n=106) excluded the 24 sequences not treated with REPLI-g, and was additionally filtered using a minimum minor allele frequency of 3% and output only a single SNP per locus.

| Dataset | *N* | Sample prep | Number of loci | Number of SNPs | Mean num. SNPs per locus | Min. depth | Max. depth | Mean depth | *Ho* |
| --- | --- | --- | --- | --- | --- | --- | --- | --- | --- |
| “WGA test” | 24 | without WGA | 1,927 | 4,637 | 2.4 | 7.9 | 40 | 21.8 | 0.15 |
|  | 21 | with WGA | 752 | 1,583 | 2.1 | 4.3 | 178 | 70* | 0.13 |
| “population genetic” | 106 | with WGA | 1,702 | 1,702 | 1 | 9.2 | 180.3 | 40.3 | 0.18 |

Table A4: Population-level WGA and non-WGA pairwise *F_ST_* calculations for the “WGA test” dataset. *F_ST_* calculations with p-values < 0.05 after a Benjamini-Hochberg correction are bolded. Locality abbreviations follow Fig. 1. The comparisons in the upper right quadrant are the same as those in the lower left, and so are not shown.

|  |  | Non-WGA | | | | WGA | | | |
| --- | --- | --- | --- | --- | --- | --- | --- | --- | --- |
|  |  | STN | NBF | TNH | LMT | STN | NBF | TNH | LMT |
| Non-WGA | STN | -- |  |  |  |  |  |  |  |
|  | NBF | **0.10** | -- |  |  |  |  |  |  |
|  | TNH | 0.01 | **0.12** | -- |  |  |  |  |  |
|  | LMT | 0.01 | **0.10** | **0.03** | -- |  |  |  |  |
| WGA | STN | -0.10 | **0.10** | 0.01 | 0.01 | -- |  |  |  |
|  | NBF | **0.06** | -0.06 | **0.08** | **0.05** | **0.06** | -- |  |  |
|  | TNH | 0.01 | **0.12** | -0.07 | **0.03** | 0.01 | **0.08** | -- |  |
|  | LMT | 0.00 | **0.10** | 0.03 | -0.09 | 0.00 | **0.06** | **0.03** | -- |


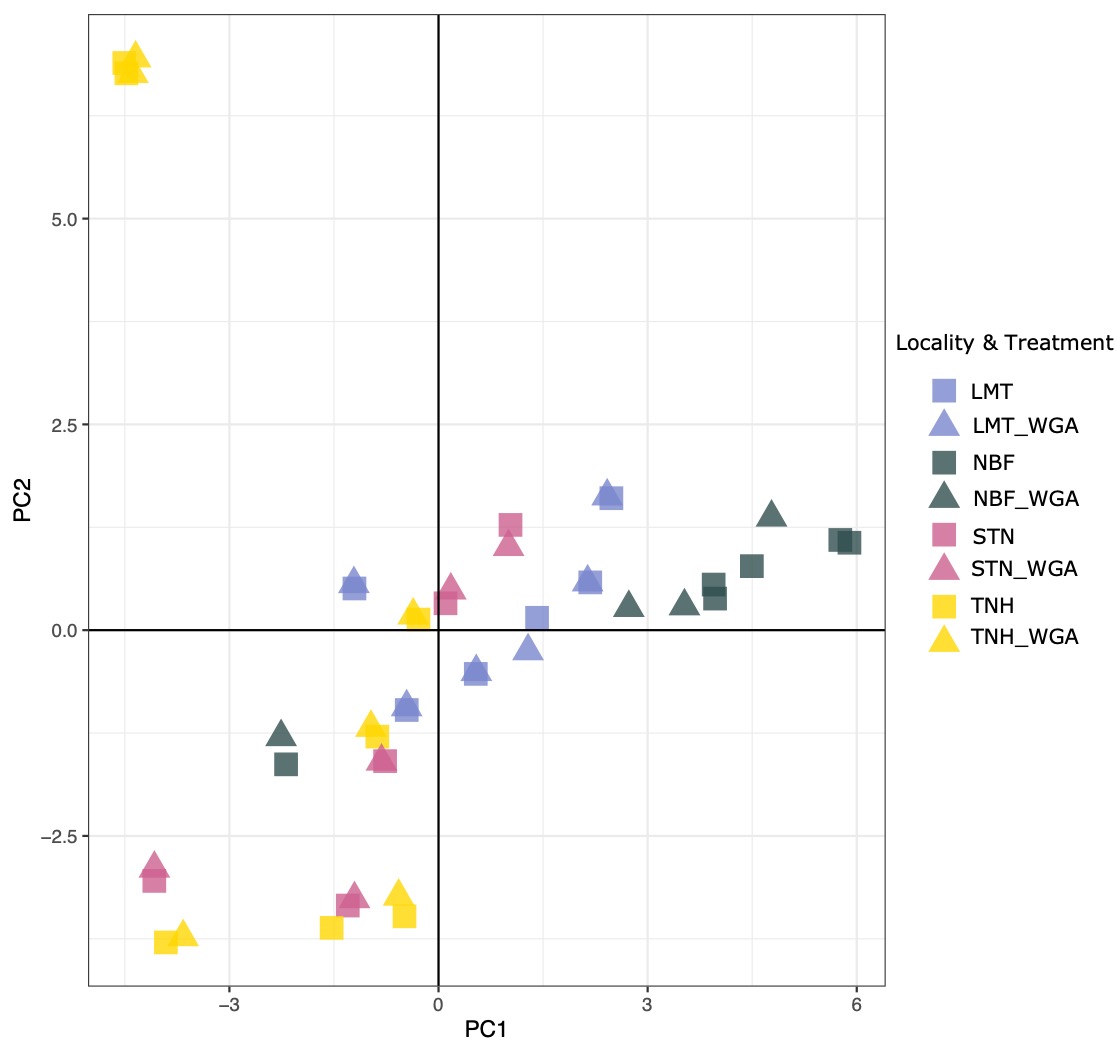


Figure A1: Principle Component Analysis of SNPs for the “WGA test” dataset shows paired clustering of individuals irrespective of WGA treatment (WGA sequences are represented in the plot by triangles, and non-WGA sequences by squares). The only exceptions to this pattern were in the NBF and TNH populations: a single WGA sequence from TNH and two from NBH were omitted from this dataset as they contained mostly missing data (see Table A3). Additionally, NBF specimens had greater than average amounts of missing data and exhibited looser clustering of paired sequences for several individuals. Locality abbreviations follow Fig. 1.
